# Metabolomic Profiling of Extracellular Vesicles from Flower and Leaf Tissues of *Hibiscus syriacus*

**DOI:** 10.3390/metabo16060386

**Published:** 2026-06-02

**Authors:** Junhe Hu, Shuting Peng, Shichang Zhou, Zhi Zeng, Shuanghui Wang, Zhenzhen Guo, Yong Chen

**Affiliations:** College of Agriculture and Biotechnology, Hunan University of Humanities, Science and Technology, Road Dingxing 7#, Loudi 417000, China

**Keywords:** *Hibiscus syriacus*, extracellular vesicles (EVs), metabolomic profiling, tissue-specific metabolism, differential metabolites (DMs), LC-MS/MS

## Abstract

**Objectives:** Plant extracellular vesicles (EVs) mediate intercellular communication and carry tissue-specific metabolites, yet tissue-resolved EV metabolomics in non-model medicinal plants remains poorly explored. *Hibiscus syriacus* is a valuable medicinal and ornamental species rich in bioactive compounds, but the metabolic profiles of flower- and leaf-derived EVs are unknown. This study aimed to characterize tissue-specific EV metabolomes of *H. syriacus* and reveal their functional implications. **Methods:** EVs were isolated from flowers (MJH) and leaves (MJY) of *H. syriacus* and verified by TEM and DLS. Untargeted LC-MS/MS metabolomics was applied to profile EV metabolites. Multivariate statistics (PCA, OPLS-DA), differential metabolite screening (VIP > 1, *p* < 0.05), and KEGG pathway enrichment were performed. **Results:** MJH- and MJY-EVs exhibited typical EV morphology and high purity. In total, 3338 metabolites were identified, dominated by lipids (29.43%). Clear metabolic separation was observed between MJH- and MJY-EVs. Thirty-nine differential metabolites were identified: 31 upregulated in MJH-EVs (lipids, pentadecanoic acid) and eight in MJY-EVs (nucleotides, secondary metabolites). Glycerophospholipid metabolism was the most enriched pathway in MJH-EVs, while MJY-EVs were linked to energy and defensive metabolism. **Conclusions:** *H. syriacus* EVs display strong tissue-specific metabolic signatures. Leaf EVs prioritize lipid metabolism for photosynthetic function and stress tolerance, while flower EVs accumulate secondary and energy-related metabolites for reproduction and defense. These findings advance plant EV biology and support potential applications of *H. syriacus* EVs in cosmetics and agriculture.

## 1. Introduction

Plant extracellular vesicles (EVs) are nanosized membrane vesicles released by plant cells into the extracellular environment [1]. Enclosed by a lipid bilayer, these EVs carry a variety of bioactive molecules, including proteins, nucleic acids, lipids, and metabolites, which enable them to participate in intercellular communication, pathogen defense, stress response, and material transport [1,2,3,4]. Plant-derived EVs exhibit antioxidant, anti-inflammatory, and antitumor activities, making them promising for biomedicine and agriculture. In recent years, plant-derived EVs have gained increasing attention due to their unique biological activities [5,6]. For example, EVs from ginger, Maca, and strawberry have been reported to exhibit antioxidant, anti-inflammatory, and antitumor effects [1,7,8], making them promising candidates for natural product development. While EV studies in model plants have advanced rapidly, plant EV metabolomics remains largely underexplored, especially regarding tissue-specific EV cargo and its biological relevance in non-model medicinal plants.

*H. syriacus*, belonging to the Malvaceae family, is a deciduous shrub with high ornamental and medicinal value [9,10]. Its flowers, leaves, and roots have been used in traditional medicine to treat hypertension, inflammation, and microbial infections [11,12]. Previous studies have confirmed that *H. syriacus* can secrete EVs from different tissues; however, the metabolic divergence of EV contents across tissues (flower vs. leaf) remains largely uncharacterized. Critically, *H. syriacus* secretes EVs from different tissues, yet tissue-specific EV metabolomes and their links to organ function remain completely unknown.

Plants exhibit significant tissue-specific metabolic characteristics due to functional differentiation: flowers are often involved in reproduction and stress resistance, while leaves are the main sites of photosynthesis and material synthesis [13]. These functional differences may lead to distinct metabolic profiles of EVs derived from different tissues, which directly affect their biological activities and application potential. Metabolomics, as a key omics technology, aims to systematically identify and quantify all metabolites in biological samples, providing comprehensive insights into metabolic pathways and biological functions [14,15,16]. Untargeted metabolomics allows for the detection of a wide range of metabolites without prior knowledge, making it suitable for discovering tissue-specific metabolic differences [17]. In plant research, metabolomics has been widely applied to characterize tissue-specific metabolite patterns in model plants such as Arabidopsis thaliana and Medicago truncatula [18]. While several studies have profiled metabolites in plant extracellular vesicles (EVs) from species including rice, grape, tomato, and Panax ginseng, most have focused on a single tissue or whole-plant extracts [19,20,21]. Tissue-specific EV metabolomics in non-model medicinal plants remains largely unexplored, highlighting the novelty and significance of the present study.

While EV research has advanced in model plants, tissue-specific EV metabolomics in non-model medicinal plants remains largely unexplored. This study aims to: (1) characterize metabolomic profiles of EVs from Hibiscus syriacus flower and leaf; (2) identify tissue-specific DMs; and (3) annotate functional pathways and predict physiological roles. We hypothesize that EV metabolic cargo matches tissue functional differentiation, providing a foundation for targeted applications of H. syriacus EVs. This work not only advances plant EV metabolomics in non-model medicinal plants but also provides a theoretical basis for the targeted development of *H. syriacus* EVs in cosmetics, agriculture, and natural products.

## 2. Materials and Methods

### 2.1. Reagents and Instruments

Methanol (CAS: 67-56-1), acetonitrile (CAS: 75-05-8), and formic acid (CAS: 64-18-6) were of chromatographic grade (Sigma-Aldrich, St. Louis, MO, USA). Ultrapure water was prepared using a Milli-Q system (Millipore, Billerica, MA, USA). Internal standards (L-2-chlorophenylalanine, CAS: 10361-88-3) were purchased from Tokyo Chemical Industry (Tokyo, Japan). Instruments were used as follows: Ultra-high-performance liquid chromatography (UPLC) system—Vanquish (Thermo Scientific, Waltham, MA, USA); Tandem mass spectrometry (MS/MS) system—Q Exactive HF-X (Thermo Scientific, Waltham, MA, USA); centrifuge—5424R (Eppendorf, Hamburg, Germany); Ultracentrifuge—Optima XE-90 (Beckman Coulter, Brea, CA, USA); Constant temperature metal mixer—MU-G02-0448 (Hangzhou Mio Instrument Co., Ltd., Hangzhou, China); Ten-thousandth electronic balance—MS105DΜ (Mettler Toledo, Zurich, Switzerland); centrifugal concentrator—CentriVap (LABCONCO, Kansas City, MO, USA); Ultrasonic cleaner—KQ5200E (Kunshan Ultrasonic Instrument Co., Ltd., Kunshan, China); Pipette—Research plus (Eppendorf, Hamburg, Germany); Automated workstation—Biomek i5 (Beckman Coulter, Brea, CA, USA); and Film sealer—Mini HES (Monad, Suzhou, China). All instruments were regularly calibrated and maintained according to manufacturer specifications to ensure optimal performance.

### 2.2. H. syriacus Material and EV Isolation

**Figure 1:**
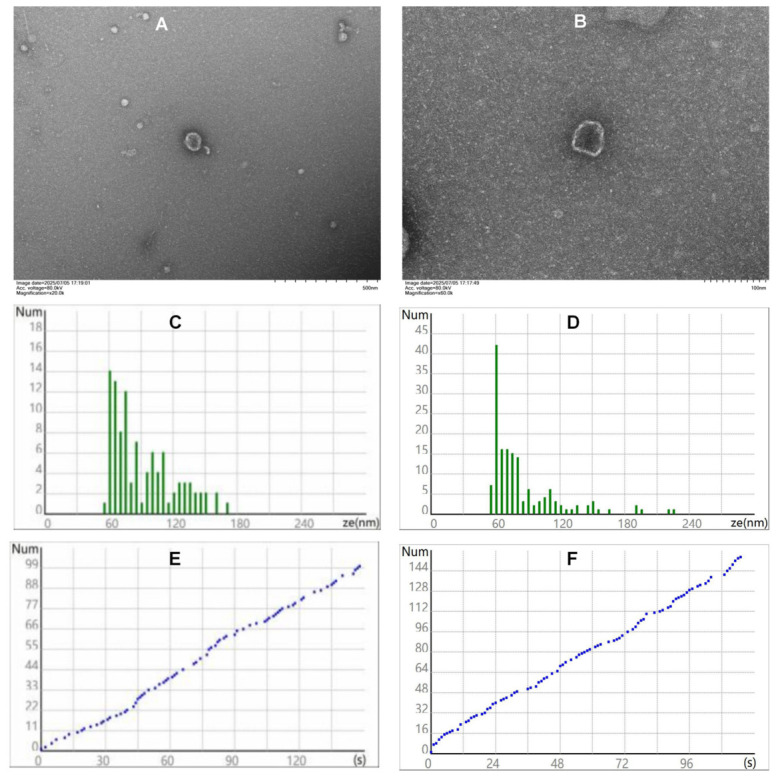
Characterization and size distribution of extracellular vesicles (EVs) isolated from Hibiscus syriacus flowers (MJH) and leaves (MJY)**.** (**A**) Transmission electron microscopy (TEM) image of flower-derived EVs (MJH), scale bar = 500 nm. (**B**) TEM image of leaf-derived EVs (MJY), scale bar = 100 nm. (**C**) Dynamic light scattering (DLS) size distribution of MJH EVs (mean diameter: 75.2 ± 8.5 nm). (**D**) DLS size distribution of MJY EVs (mean diameter: 90 ± 29.0 nm). Nanoparticle tracking analysis (NTA) cumulative concentration curves, showing the total particle count. The final concentration was 1.06 × 10^9^ particles/mL for MJH-EVs (**E**), and 2.26 × 10^9^ particles/mL for MJY-EVs (**F**), confirming high vesicle yields and monodisperse populations.

Healthy and mature flowers (MJH) and leaves (MJY group) were harvested at 8:00–9:00 AM in Ziyang County, Yiyang City, Hunan Province, to minimize physiological variation. Each group comprised three biological replicates, each consisting of 50 g of flowers or leaves, resulting in a total of 6 samples (3 biological replicates per group). The experimental design and procedure are shown in Figure 1. Samples were initially rinsed thoroughly with distilled water, and surface moisture was removed by blotting with filter paper. All operations were conducted in a biosafety cabinet. The samples were disinfected with 75% ethanol for 1 min, subsequently peeled, and juiced using a juicer; in cases where juice volume was insufficient, a suitable volume of phosphate-buffered saline (PBS) was added for dilution. The resulting mixture was transferred to a fresh centrifuge tube and centrifuged at 2000× *g* for 30 min at 4 °C.

Subsequently, the supernatant was carefully transferred to a new centrifuge tube and re-centrifuged at 10,000× *g* for 45 min at 4 °C to eliminate larger EVs. The supernatant was collected and filtered through a 0.45 μm membrane, with the filtrate retained. The filtrate was then transferred to a fresh centrifuge tube and subjected to ultracentrifugation at 100,000× *g* for 70 min at 4 °C. After ultracentrifugation, the supernatant was discarded, and the pellet was resuspended in 10 mL of pre-chilled 1 × PBS. Ultracentrifugation was repeated under identical conditions (100,000× *g*, 4 °C, 70 min). Finally, the supernatant was discarded, the final pellet was resuspended in 100 μL of pre-chilled 1 × PBS, and the preparation was stored at −80 °C.

### 2.3. Sample Extraction of EVs

Vesicle samples were extracted via a modified methanol–water method, as described by [19], with the detailed procedures as follows: Briefly, all vesicle samples were first lyophilized using a freeze dryer (Labconco, USA). Subsequently, 1000 μL of 80% methanol–water solution containing L-2-chlorophenylalanine (internal standard, final concentration 1 μg/mL) was added to the lyophilized samples, followed by vortexing for 3 min. The centrifuge tubes containing the mixture were then subjected to a freeze–thaw–vortex cycle repeated three times: each cycle included quick freezing in liquid nitrogen for 5 min, thawing on dry ice for 5 min, subsequent thawing on ice for 5 min (excessive temperature difference was avoided to prevent tube cap explosion and sample ejection), and vortexing for 2 min to ensure homogeneous mixing. After the cycles, the mixtures were centrifuged at 12,000 r/min for 10 min at 4 °C, and all the resulting supernatants were transferred into correspondingly numbered centrifuge tubes and concentrated to dryness using a centrifugal concentrator. The dried residues were reconstituted with 200 μL of 70% methanol–water solution, vortexed for 3 min, and sonicated in an ice bath for 10 min. A further centrifugation step was performed at 12,000 r/min for 3 min at 4 °C, and 80 μL of the final supernatants was transferred into the inner liners of corresponding injection vials for subsequent UPLC-MS/MS analysis. For quality control (QC) purposes, QC samples were prepared by mixing equal volumes (10 μL) of supernatants from all individual samples, which were used to monitor the stability and reliability of the entire analytical system throughout the experiment.

### 2.4. UPLC-MS/MS Analysis Conditions

Ultra-performance liquid chromatography (UPLC) separation was performed on a Waters ACQUITY Premier HSS T3 column (1.8 µm, 2.1 mm × 100 mm; Waters, Milford, MA, USA). The mobile phase consisted of solvent A (0.1% formic acid in ultrapure water) and solvent B (0.1% formic acid in acetonitrile). The column temperature was maintained at 40 °C with a constant flow rate of 0.4 mL/min, and the injection volume was set at 3 μL. A gradient elution program was applied as follows: initial conditions were held at 95% A and 5% B for 0.0 min; linearly adjusted to 80% A and 20% B at 1.0 min; further changed to 1% A and 99% B at 3.0 min and held until 4.5 min; rapidly returned to the initial ratio (95% A, 5% B) at 4.6 min; and finally equilibrated for 1.4 min to reach a total run time of 6.0 min.

Mass spectrometric detection was carried out using a Q Exactive HF-X mass spectrometer (Thermo Scientific, Waltham, MA, USA) equipped with both positive (ESI+) and negative (ESI−) electrospray ionization modes. The key ionization and detection parameters were optimized as follows: ionization voltage was set at 3800 V for ESI+ and 3400 V for ESI−; sheath gas and auxiliary gas flow rates were fixed at 60 Arb and 20 Arb, respectively, for both ionization modes. The ion transfer tube temperature was maintained at 320 °C for both ESI+ and ESI−, while the atomization temperature was set at 350 °C for ESI+ and 650 °C for ESI−. For MS1 scans, the mass range was 70–1000 Da, with a resolution of 60,000 and an automatic gain control (AGC) target of 3.00 × 10^6^ for both ionization modes. The collision energy step was set at 50 V. For MS2 scans, the mass range was also 70–1000 Da with a resolution of 15,000 and an AGC target of 1.00 × 10^5^. Collision energy (CE) was applied at three levels (30, 40, 50 V) for both ESI+ and ESI−. The intensity threshold for triggering MS2 scans was 1.00 × 10^6^ cps, and the maximum number of candidate ions selected for MS2 fragmentation was 10 per cycle, with a dynamic exclusion time of 3 s.

### 2.5. Data Processing and Quality Control

Raw MS data were converted to mzML format using ProteoWizard software 3.0, followed by peak extraction, alignment, and retention time correction using XCMS software 3.7.1 [20]. Peaks with a missing rate > 50% in each group were filtered out. Missing values were filled using the K-nearest neighbor (KNN) method (for missing rate < 50%) and the 1/5 minimum value method (for a missing rate > 50%). Peak areas were corrected using the support vector regression (SVR) method to reduce matrix effects.

Metabolite identification was performed by searching against a self-built laboratory database, integrated public databases (HMDB, Metlin, PubChem), and prediction databases. Metabolites with a comprehensive identification score > 0.5 and a CV value < 0.3 in QC samples were retained. Finally, positive and negative mode data were merged (retaining metabolites with the highest qualitative grade and smallest CV value) to obtain the final metabolomic dataset.

To ensure the reliability and stability of the analytical system, multiple quality assessment analyses were performed on the quality control (QC) samples, as detailed below: Firstly, total ion chromatogram (TIC) overlap analysis was conducted by overlapping the TIC profiles of all QC samples, which was aimed at evaluating the repeatability of metabolite extraction and detection processes. Secondly, Pearson correlation analysis was carried out to calculate the Pearson correlation coefficients between pairwise QC samples; the analytical system was considered stable when the absolute value of the correlation coefficient (|r|) exceeded 0.99. Additionally, the distribution of coefficient of variation (CV) values of all metabolites in QC samples was analyzed using the empirical cumulative distribution function (ECDF). A QC sample was regarded to have excellent data stability if more than 75% of its metabolites exhibited a CV value less than 0.3. Furthermore, internal standard stability analysis was performed by calculating the CV values of the internal standard (L-2-chlorophenylalanine) in QC samples; a CV value less than 0.2 indicated the reliability of the entire extraction and detection process.

Metabolomic data were subjected to multivariate statistical analyses to characterize metabolic differences among experimental groups, with all analyses conducted using R software (version 4.1.2). First, unsupervised principal component analysis (PCA) was performed via the prcomp function with unit variance (UV) scaling applied to the data; this approach was used to visually assess the overall metabolic separation and clustering patterns between groups. Subsequently, supervised orthogonal partial least squares discriminant analysis (OPLS-DA) was implemented using the MetaboAnalystR package (version 4.2.0) to enhance the discrimination between groups. Prior to OPLS-DA analysis, raw data were preprocessed with Log2 transformation and centering to optimize data distribution and reduce systematic variation. The robustness and validity of the OPLS-DA model were verified by a permutation test with 200 permutations; model stability and predictive ability were evaluated using R2X (explained variance of X), R2Y (explained variance of Y), and Q2 (predictive ability) values, with a Q2 value > 0.5 considered indicative of a reliable and valid model. Finally, differential metabolites between groups were screened based on two stringent criteria: a variable importance in projection (VIP) value > 1 (derived from the OPLS-DA model) and a *p*-value < 0.05 (determined by Student’s *t*-test).

### 2.6. Functional Annotation and Enrichment Analysis

For functional annotation, all confidently identified metabolites were annotated against three widely recognized, authoritative databases: the Kyoto Encyclopedia of Genes and Genomes (KEGG) Compound Database (https://www.genome.jp/kegg/compound/, last updated March 2024, accessed on 1 October 2025), the Human Metabolome Database (HMDB, https://www.hmdb.ca/hml, Version 5.3, accessed on 1 October 2025), and the LIPID MAPS Structure Database (LMSD, https://www.lipidmaps.org/, Version 4.0, accessed on 1 October 2025). Metabolite identifiers derived from LC-MS/MS raw data were systematically converted to corresponding KEGG, HMDB, and LMSD IDs via batch database matching. Only metabolites with high-confidence matches (match score ≥ 0.85) were retained for subsequent analyses to ensure data reliability. To identify biological pathways significantly enriched with DMs between flower-derived EVs (MJY-EVs) and leaf-derived EVs (MJH-EVs), KEGG pathway enrichment analysis was conducted using two complementary approaches, MetaboAnalyst 5.0 online platform ([https://www.metaboanalyst.ca/] and R package clusterProfiler [Version 4.4.4], accessed on 1 October 2025), for cross-validation to enhance result robustness and reliability. Statistical significance was determined via Fisher’s exact test, with raw *p*-values adjusted using the false discovery rate (FDR) method to reduce false positives. Pathways were considered significantly enriched if they met the following criteria: adjusted *p*-value (FDR) < 0.05 and Rich Factor > 0.1. The Rich Factor is the ratio of DMs mapped to a KEGG pathway to the total study-identified metabolites annotated to that pathway in KEGG, quantifying DM enrichment magnitude per pathway. Furthermore, to investigate potential regulatory relationships and co-occurrence patterns among key DMs, pairwise correlation analysis was conducted using the R package corrplot (Version 0.92). Pearson correlation coefficients were computed for all DM pairs, and correlations were deemed statistically significant at a threshold of *p* < 0.001. Visualization of enrichment results (bubble plots and bar charts) and correlation analysis outcomes (heatmaps) was generated using the R package ggplot2 (Version 3.4.2), with representative plots displayed respectively. Unless explicitly stated otherwise, all software parameters were maintained at their default settings to ensure full reproducibility of the analytical workflow.

### 2.7. Statistical Analysis

For two-group analysis, differential metabolites were determined by the VIP (VIP > 1) and *p*-value (*p*-value < 0.05, Student’s *t* test). VIP values were extracted from OPLS-DA results, which also contain score plots and permutation plots, and were generated using the R package MetaboAnalystR. The data were log transformed (Log2) and mean centered before OPLS-DA. In order to avoid overfitting, a permutation test (200 permutations) was performed. A *p*-value < 0.05 was considered statistically significant unless otherwise specified.

## 3. Results

### 3.1. Metabolomic Profiling of EVs

#### 3.1.1. Isolation and Characterization of EVs

Transmission electron microscopy (TEM) analysis revealed EVs with a spherical or cup-shaped morphology and a distinct lipid bilayer membrane, consistent with exosome-like structural features (Figure 1A–D). Representative micrographs (Figure 1A,B) revealed the presence of membrane-bound EVs with a characteristic cup-shaped morphology, consistent with the structural features of exosomes and exosome-like EVs reported in plant and animal systems. The EVs exhibited a heterogeneous size distribution, with diameters ranging from approximately 50 nm to 200 nm, as indicated by the scale bars in the TEM images (Figure 1A: 500 nm; Figure 1B: 100 nm). Dynamic light scattering (DLS) indicated that the mean diameters are 75.2 ± 8.5 nm for flower tissue (MJH) EVs (Figure 1C) and 90 ± 29.0 nm for leaf tissue (MJY) EVs (Figure 1D), which aligns with the size range observed via TEM. The final particle concentrations, as determined by NTA, were 1.06 × 10^9^ particles/mL for MJH-EVs and 2.26 × 10^9^ particles/mL for MJY-EVs (Figure 1E,F), confirming high yields and monodisperse vesicle populations.

#### 3.1.2. Quality Control and Multivariate Statistical Analysis of Data

**Figure 2:**
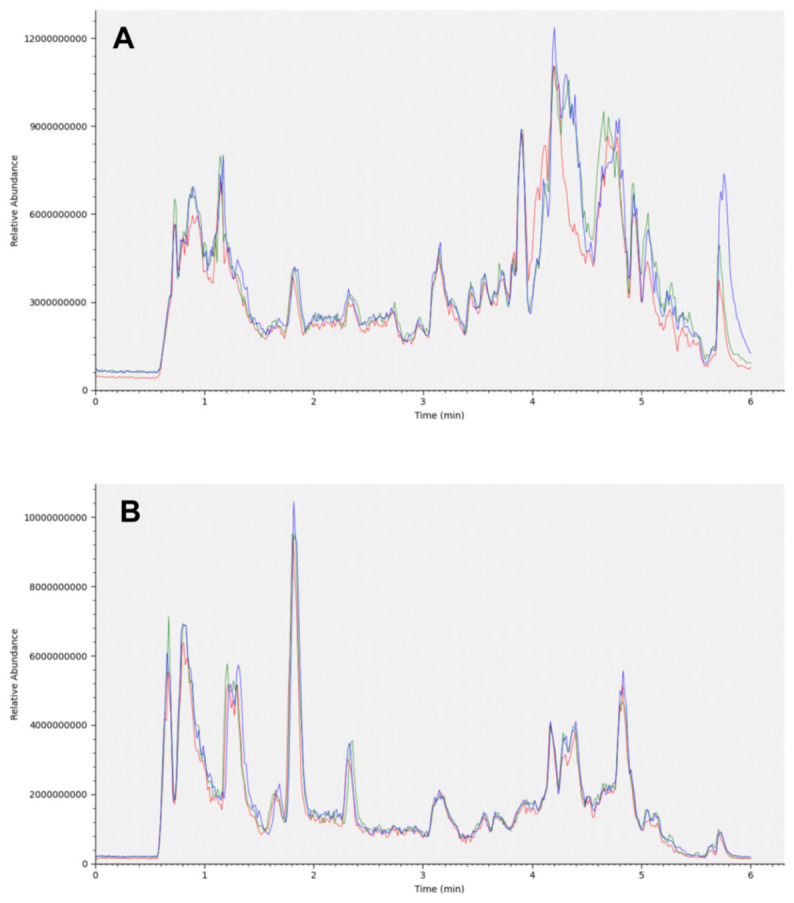
Total ion current (TIC) overlap of quality control (QC) samples. The results demonstrate high overlap in the total ion current curves of metabolite detection in the negative ion mode (**A**) and positive ion mode (**B**), indicating consistent retention times and peak intensities. This suggests that the mass spectrometer maintains good signal stability when analyzing the same sample at different time points. The instrument’s high stability provides crucial assurance for data repeatability and reliability. Note: The overlapping curves indicate consistent retention times and peak intensities of metabolites in QC samples.

The TIC overlap diagram of quality control (QC) samples showed high overlap of total ion flow curves in both negative (Figure 2A) and positive ion modes (Figure 2B), indicating consistent retention times and peak intensities of metabolites, and good repeatability of the extraction and detection processes. By overlaying and analyzing the total ion current (TIC) profiles obtained from mass spectrometric detection of different QC samples, the reproducibility of metabolite extraction and detection—i.e., technical reproducibility—can be evaluated. The high stability of the instrument provides critical assurance for the repeatability and reliability of the data. The CV values of four internal standards in QC samples ranged from 0.0125 to 0.2283 (Table 1), all <0.3, indicating stable recovery of metabolites during extraction and detection. ECDF analysis of CV values showed that 82% of metabolites in QC samples had CV < 0.3, and 91% had CV < 0.5 (Table 1), meeting the requirements of metabolomic analysis for data stability. The raw LC-MS/MS metabolomics data have been deposited in a public repository (https://ngdc.cncb.ac.cn/omix/releaseList, accessed on 22 May 2026) with a unique accession number (OMIX016970).

**Figure 3:**
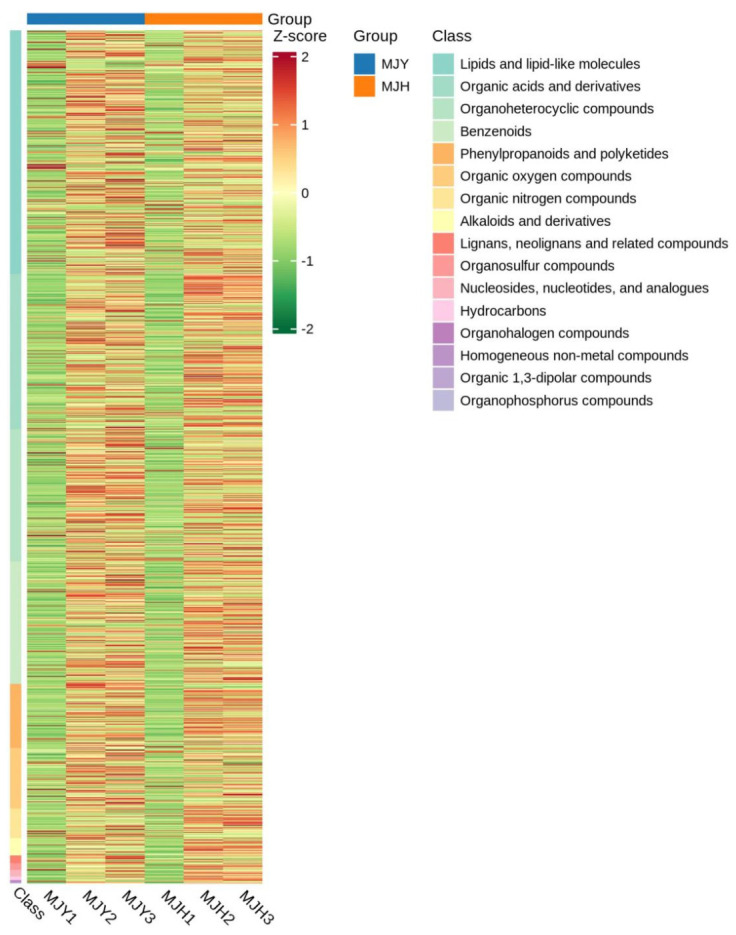
Hierarchical clustering heatmap of metabolites in flower (MJH) and leaf (MJY) EVs. Metabolite abundances were normalized by Z-score, with color intensity representing relative expression levels: red indicates metabolites with Z-score ≥ 1 (upregulated), green indicates Z-score ≤ −1 (downregulated), and yellow indicates near-baseline expression (Z-score ≈ 0). Rows represent individual metabolites, annotated by their metabolic class (right panel), with lipids and lipid-like molecules constituting the most abundant category. Columns denote biological replicates of leaf EVs (MJY1–3) and flower EVs (MJH1–3). Hierarchical clustering reveals distinct metabolic signatures between floral and foliar EVs, highlighting tissue-specific enrichment of lipid metabolites, phenylpropanoids, and organic acids, which align with functional specialization in inter-tissue signaling and stress resilience.

To validate the reliability of the metabolomic data, unsupervised hierarchical clustering was performed on Z-score-normalized metabolite abundances from EVs of *H. syriacus* flowers (MJH1-3) and leaves (MJY1-3), as shown in Figure 3. As shown in the heatmap (Figure 3), biological replicates within the flower group (MJH) and leaf group (MJY) clustered tightly, while the two tissue groups were clearly separated. This distinct clustering pattern indicates high intra-group reproducibility and robust inter-group metabolic divergence, confirming the consistency of sample processing and analytical workflows. A total of 3338 metabolites were annotated and categorized into 16 functional classes based on chemical taxonomy (Figure 3). The most prevalent classes included: lipids and lipid-like molecules, organic acids and derivatives, and organoheterocyclic compounds (Figure 3). These classes collectively accounted for a large proportion of the detected metabolome, and other notable classes included benzenoids, phenylpropanoids and polyketides, and organic oxygen compounds, reflecting the metabolic diversity of EVs in *H. syriacus* flowers and leaves (Figure 3).

**Figure 4:**
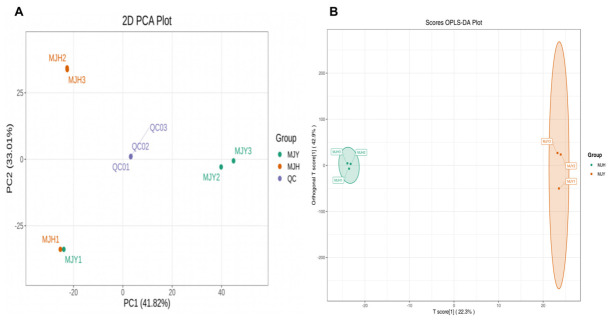
Multivariate statistical analysis of metabolic profiles in EVs from MJH group versus MJY group. (**A**). Two-dimensional principal component analysis (PCA) score plot, where PC1 and PC2 explain 41.82% and 33.01% of total metabolic variance, respectively. Green dots represent leaf EV replicates (MJY1–3), orange dots represent floral EV replicates (MJH1–3), and purple dots denote quality control (QC) samples. The tight clustering of QC samples confirms analytical stability and reproducibility, while partial separation of MJY and MJH groups indicates inherent metabolic differences between leaf and floral EVs. (**B**). Orthogonal projections to latent structures discriminant analysis (OPLS-DA) score plot, which maximizes separation between groups by filtering orthogonal noise. The clear segregation of MJY (orange) and MJH (green) clusters, enclosed within 95% confidence ellipses, demonstrates significant metabolic divergence between leaf and floral EV cargo, supporting the presence of tissue-specific functional specialization in EV-mediated metabolite trafficking.

**Figure 5:**
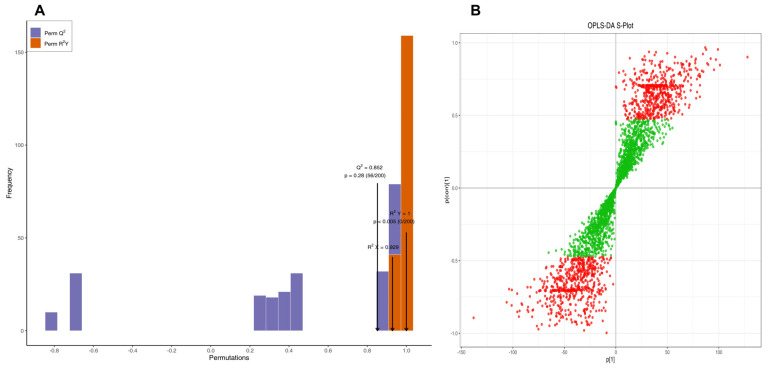
Permutation testing analysis of metabolic profiles in EVs from MJH group versus MJY group. (**A**) Permutation test (200 iterations) showing the distribution of R^2^Y (orange) and Q^2^ (blue) values. The true model values (R^2^Y = 1, Q^2^ = 0.852) are significantly higher than those obtained from permuted datasets (*p* = 0.008 for R^2^Y, *p* = 0.28 for Q^2^), indicating no overfitting. (**B**) OPLS−DA S-plot showing the contribution and reliability of each metabolite to the group separation. Points in the upper right (red) and lower left (red) corners represent metabolites with high covariance and correlation, which are the most influential variables distinguishing the MJH and MJY groups.

Multivariate statistical analysis enables the “simplification and dimensionality reduction” of high-dimensional complex data while preserving the original information to the greatest extent, thereby establishing reliable mathematical models to summarize and characterize the metabolic profiles of the study subjects. Multivariate statistical analysis revealed clear metabolic divergence between MJH and MJY. In the 2D PCA score plot (Figure 4A), PC1 (41.82%) and PC2 (33.01%) together explained 82.85% of the total variance, with biological replicates clustering within their respective groups, indicating high reproducibility of the metabolomic data. The OPLS-DA score plot (Figure 4B) further enhanced group separation along the predictive component (T score [1], 22.3%) and orthogonal component (Orthogonal T score [1], 42.9%), with 95% confidence ellipses confirming robust discrimination between floral and leaf vesicle samples. The OPLS-DA model exhibited a high goodness-of-fit (R^2^Y = 1) and excellent predictive ability (Q^2^ = 0.852), indicating robust metabolic differences between the two groups. To assess overfitting, a permutation test with 200 iterations was performed (Figure 5A). The true model’s R^2^Y was significantly higher than permuted values (*p* = 0.008), while the Q^2^ value did not meet the conventional significance threshold (*p* = 0.28). However, the negative intercept of the Q^2^ regression line confirms the absence of overfitting, supporting the reliability of the model. The OPLS-DA S-plot (Figure 5B) facilitated identification of discriminant metabolites by plotting correlation (R [1]) against significance (p(corr) [1]). Metabolites distant from the origin exhibited strong predictive power, with red dots (MJY) and green dots (MJH) highlighting tissue-specific metabolites that drive the observed metabolic differences.

#### 3.1.3. Metabolomic Profiling of EVs from Different Tissues

**Figure 6:**
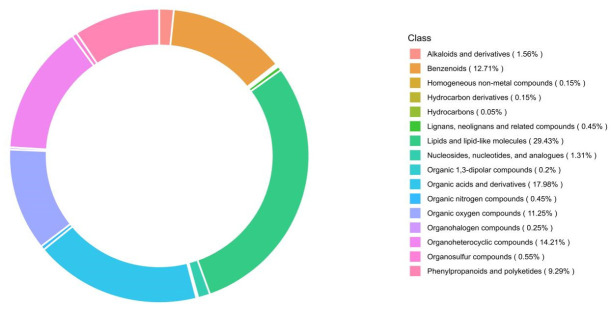
Circular bar chart of metabolite class distribution in EV sample. The chart displays the relative abundance (percentage) of all identified metabolites categorized by their chemical class, with the right panel providing a color-coded legend for each category. Lipids and lipid-like molecules (29.43%) dominate the EV cargo, followed by organic acids and derivatives (17.98%) and organoheterocyclic compounds (14.21%). Other notable classes include benzenoids (12.71%), organic oxygen compounds (11.25%), and phenylpropanoids and polyketides (9.29%), while low-abundance classes (hydrocarbons, 0.05%) reflect selective enrichment of bioactive metabolites.

A total of 3338 metabolites were identified in the two groups of samples, including 1586 in positive ion mode and 1752 in negative ion mode. Among them, 2989 metabolites were successfully annotated by secondary mass spectrometry, covering 16 categories such as lipids and lipid-like molecules, organic acids and derivatives, organoheterocyclic compounds, and phenylpropanoids and polyketides (Figure 6). To characterize the overall metabolic landscape of the samples, we first analyzed the relative abundance of all identified metabolites across their respective chemical classes (Figure 6). The circular ring chart reveals a highly heterogeneous distribution, with lipids and lipid-like molecules being the most dominant class, accounting for 29.43% of the total detected metabolites. This was followed by organic acids and derivatives (17.98%), organoheterocyclic compounds (14.21%), and benzenoids (12.71%), together comprising over 74% of the total metabolome. Intermediate abundance classes included organic oxygen compounds (11.25%) and phenylpropanoids and polyketides (9.29%). In contrast, several metabolite classes were detected only at trace levels, including homogeneous non-metal compounds (0.15%), hydrocarbon derivatives (0.15%), and hydrocarbons (0.05%), each contributing less than 0.2% of the total profile. Other low-abundance classes (≤2%) included alkaloids and derivatives (1.56%), nucleosides, nucleotides, and analogs (1.31%), organosulfur compounds (0.55%), lignans, neolignans and related compounds (0.45%), organic 1,3-dipolar compounds (0.2%), and organohalogen compounds (0.25%).

#### 3.1.4. Identification of Differential Metabolites

**Figure 7:**
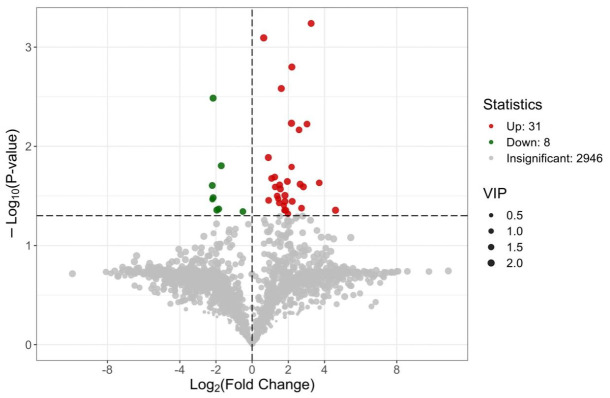
Volcano plot illustrating differential metabolite profiling in EVs from MJH group versus MJY group. The x-axis displays the log_2_(fold change) of metabolite abundance (MJH relative to MJY), while the y-axis shows the −log_10_(*p*-value) to indicate statistical significance. Horizontal and vertical dashed lines denote significance thresholds of *p* < 0.05 and ∣log2FC∣ ≥ 1, respectively. Metabolites are color-coded by their differential expression status: red dots represent 31 significantly upregulated metabolites (predominantly lipids and phenylpropanoids, concentrated in the right quadrant), green dots represent 8 significantly downregulated metabolites (including purine derivatives, clustered in the left quadrant), and gray dots represent 2946 metabolites with no significant differential abundance (forming the central and peripheral background).

To delineate the tissue-specific metabolic cargoes of extracellular vesicles (EVs) isolated from *H. syriacus* flowers (MJH group) and leaves (MJY group), we implemented an untargeted metabolomics workflow, which enabled the confident annotation of 237 unique metabolites (present in all replicates, consistent annotation), as shown in Table S1. Based on the screening criteria of VIP > 1 and *p* < 0.05, a total of 39 differential metabolites were identified between MJH and MJY groups, including 31 upregulated and eight downregulated metabolites in the MJH group compared to MJY group (Supplementary Table S2). To characterize metabolomic differences between EVs from *H. syriacus* flowers (MJY) and leaves (MJH), we performed volcano plot analysis using thresholds of variable importance in projection (VIP) > 1, *p*-value < 0.05, and |log_2_(fold change, FC)| ≥ 1, as shown in Figure 7. Of all detected metabolites, 2946 showed no significant difference (gray dots), clustering near the origin with |log_2_FC| < 1 and *p*-value ≥ 0.05, while 31 metabolites were significantly upregulated in flower-derived EVs (red dots), exhibiting elevated log_2_FC (>2), strong statistical significance and high VIP scores (up to 2.0) that underscore their role in distinguishing tissue-specific EV populations. Only eight metabolites were downregulated in leaf EVs (green dots), with negative log_2_FC (<−1) and significant *p*-values but lower VIP scores. The nearly 4:1 ratio of up- to downregulated metabolites indicates a marked bias toward metabolic upregulation in leaf-derived EVs, reflecting tissue-specific functional specialization in *H. syriacus* that may support leaf-specific physiological processes such as photosynthesis or stress responses.

**Figure 8:**
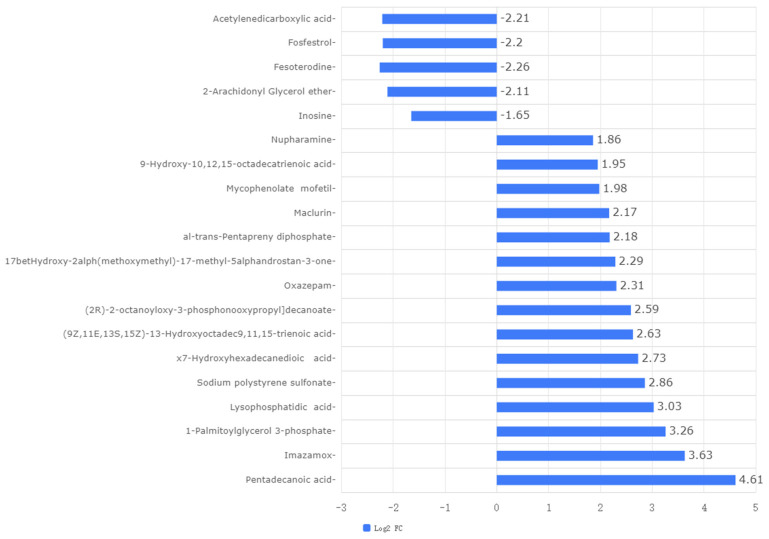
The top 20 differentially abundant metabolites in EVs of MJH group versus MJY group. The x-axis represents the log_2_(fold change) (log_2_FC) of metabolite abundance (MJH relative to MJY), where positive values indicate upregulation in floral EVs and negative values indicate downregulation. Metabolites are color-coded by their differential expression status: red bars denote 15 significantly upregulated metabolites (predominantly lipid derivatives and phenylpropanoids), with pentadecanoic acid (log_2_FC = 4.61) exhibiting the greatest magnitude of increase, followed by lysophosphatidic acid (log_2_FC = 3.03) and maclurin (log_2_FC = 2.17). Green bars denote 5 significantly downregulated metabolites, including 2-arachidonyl glycerol ether (log_2_FC = −2.11) and inosine (log_2_FC = −1.65), which are linked to purine metabolism and lipid signaling.

Multivariate statistical analysis via orthogonal partial least squares discriminant analysis (OPLS-DA) identified 68 differential metabolites (DMs) with variable importance in projection (VIP) > 1.0, which were further validated by univariate Student’s *t*-test (*p*-value < 0.05) to ensure statistical robustness. Based on the OPLS-DA model, the top 10 differential metabolites with the highest VIP values were identified between the MJH (flower EVs) and MJY (leaf EVs) groups, including propamocarb (VIP = 2.11), maclurin (VIP = 2.08), pentadecanoic acid (VIP = 1.99), monoethyl carbonate (VIP = 1.99), and narirutin (VIP = 1.94) (Table 2). Most of these metabolites were significantly upregulated in flower-derived EVs, with pentadecanoic acid showing the highest fold change (log_2_FC = 4.61). Functional annotation indicated that these metabolites are mainly involved in lipid metabolism, secondary metabolism, and organic acid metabolism, reflecting the distinct metabolic characteristics of flower EVs. The log_2_FC values of metabolites upregulated in leaf-derived EVs ranged from 1.86 to 4.61, while those of metabolites downregulated in leaf-derived EVs (i.e., enriched in flower-derived EVs) ranged from −2.21 to −1.82 (Figure 8), indicating substantial differences in their abundance between the two EV populations. Among the metabolites upregulated in leaf-derived EVs, pentadecanoic acid exhibited the most marked enrichment, with a log_2_FC of 4.61, followed by 1-palmitoylglycerol 3-phosphate (log_2_FC of 3.24). A total of 14 metabolites were significantly upregulated in leaf-derived EVs, with log_2_FC values spanning from 1.86 to 4.61, as shown in Figure 8. In contrast, five metabolites were significantly enriched in flower-derived EVs, with acetylenedicarboxylic acid showing the most marked differential accumulation (log_2_FC of −2.21). Other notable flower-enriched metabolites included 2-arachidonyl glycerol ether (log_2_FC of −2.03), with log_2_FC values ranging from −2.21 to −1.82, as shown in Figure 8. These DMs represent key metabolic signatures that distinguish leaf-derived EVs from flower-derived EVs, providing a foundation for further functional characterization of tissue-specific extracellular vesicle metabolism in *H. syriacus*.

**Figure 9:**
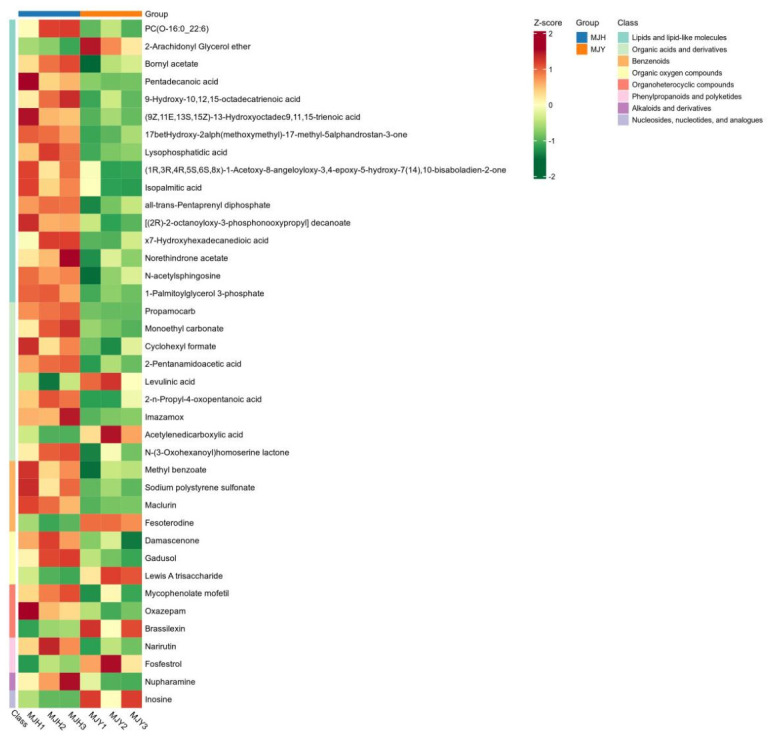
Hierarchical clustering heatmap of differential metabolite profiles in EVs from MJH group versus MJY group. Metabolite abundances were normalized by Z-scores, with color intensity representing relative expression levels: red indicates metabolites with Z-score ≥ 1 (upregulated in floral EVs), green indicates Z-score ≤ −1 (downregulated in floral EVs), and yellow indicates near-baseline expression (Z-score ≈ 0). Rows represent 39 key differential metabolites (annotated by name and metabolic class via the right-hand legend, where lipids and lipid-like molecules constitute the most abundant category), and columns denote biological replicates of leaf EVs (MJY1–3) and floral EVs (MJH1–3). Hierarchical clustering reveals clear segregation of leaf and floral EV samples, with distinct metabolic signatures: floral EVs exhibit upregulation of lipid derivatives (pentadecanoic acid, lysophosphatidic acid) and phenylpropanoids (maclurin), while leaf EVs show higher levels of purine metabolites (inosine) and lipid signaling molecules (2-arachidonyl glycerol ether).

Hierarchical clustering analysis (HCA), visualized as a Z-score-normalized heatmap (Figure 9), revealed a clear separation between MJH and MJY EV metabolomes: metabolites including PC(O-16:0_22:6), 2-arachidonyl glycerol ether, and pentadecanoic acid were markedly upregulated in flower-derived EVs, while levulinic acid and acetylenedicarboxylic acid exhibited preferential enrichment in leaf-derived EVs. Taxonomic classification of DEMs demonstrated that the largest proportion belonged to “lipids and lipid-like molecules” (lysophosphatidic acid, 1-palmitoylglycerol 3-phosphate), followed by “organic acids and derivatives” (9-hydroxy-10,12,15-octadecatrienoic acid) and “benzenoids” (methyl benzoate). Notably, the enrichment of phosphatidylcholines and glycerol ethers in flower EVs suggests a potential role in mediating floral reproductive signaling or membrane remodeling during flower development, whereas the accumulation of organic acids linked to primary carbon metabolism in leaf EVs points to their involvement in foliar stress responses or nutrient homeostasis. Collectively, these findings establish that *H. syriacus* EVs carry distinct, tissue-specific metabolite profiles, which likely underpin the functional specialization of EVs in coordinating physiological processes across floral and foliar tissues.

#### 3.1.5. Functional Annotation and Pathway Enrichment Analysis of Differential Metabolites

**Figure 10:**
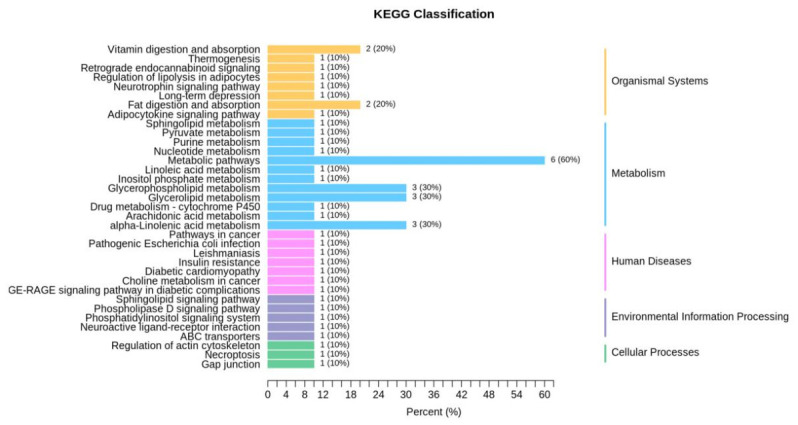
KEGG pathway classification of annotated metabolites in EVs from *H. syriacus* flowers and leaves. The x-axis displays the percentage of metabolites mapped to each KEGG pathway category, with color-coded bars representing higher-level pathway classes: metabolism (blue, 60%), Organismal Systems (orange, 20%), Environmental Information Processing (purple), and Cellular Processes (green). Within the metabolism category, core sub-pathways include metabolic pathways (60%), glycerolipid metabolism (30%), glycerophospholipid metabolism (30%), and alpha-linolenic acid metabolism (30%), reflecting the dominance of lipid metabolic processes in EV cargo. The Organismal Systems pathways (20%) include vitamin and fat digestion/absorption, indicating potential nutrient transport functions.

To further dissect the biological relevance of the identified differential metabolites (DEMs) in extracellular vesicles (EVs) from *H. syriacus* flowers and leaves, we conducted Kyoto Encyclopedia of Genes and Genomes (KEGG) pathway enrichment analysis. The results, visualized in Figure 10, showed that DMs were annotated to five major functional categories: metabolism, Organismal Systems, Human Diseases, Environmental Information Processing, and Cellular Processes. Notably, the “Human Diseases” and “Organismal Systems” categories represent general pathway annotations in the KEGG database and do not indicate direct biological functions in plants. Notably, the largest proportion of DEMs was assigned to the metabolism category (60%), with highly represented pathways including “metabolic pathways” (60%), “glycerophospholipid metabolism” (30%), “glycerolipid metabolism” (30%), and “alpha-linolenic acid metabolism” (30%), highlighting the enrichment of lipid metabolic processes that align with the tissue-specific accumulation of lipid species in flower EVs. The Organismal Systems category (20%) included pathways such as “Vitamin digestion and absorption” (20%) and “fat digestion and absorption” (20%), while smaller proportions of DEMs were mapped to “Cellular Processes” (“Regulation of actin cytoskeleton”). Pathways under “Organismal Systems” and “Human Diseases” were annotated due to conserved enzyme reactions across species, rather than reflecting plant-specific physiological roles. These findings indicate that the differential metabolite cargoes in EVs from floral and foliar tissues are functionally linked to tissue-specific physiological processes, with lipid metabolic pathways in flower EVs potentially supporting reproductive development and primary metabolic pathways in leaf EVs underpinning nutrient homeostasis and stress responses. Collectively, KEGG enrichment analysis contextualizes the metabolic divergence of EVs, reinforcing their role in mediating tissue-specific biological functions in *H. syriacus*.

**Figure 11:**
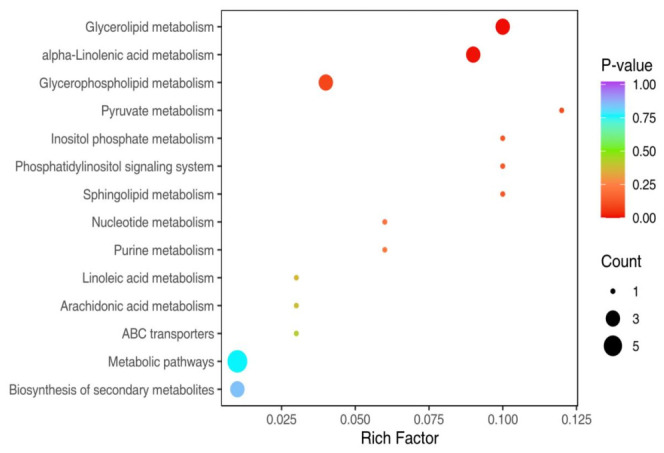
KEGG pathway enrichment analysis of differential metabolites between MJH-EVs and MJY-EVs. The bubble plot displays significantly enriched KEGG metabolic pathways. The x-axis represents the Rich Factor (ratio of the number of differential metabolites mapped to the pathway to the total number of metabolites in that pathway). The color of each bubble indicates the *p*-value (redder colors correspond to smaller *p*-values, indicating higher significance), and the size of the bubble reflects the count of differential metabolites in the pathway. The most significantly enriched pathways include glycerolipid metabolism, alpha-linolenic acid metabolism, and glycerophospholipid metabolism.

Among the 39 differential metabolites, 23 were successfully annotated in the KEGG database, involving 10 metabolic pathways (Table 3). The top five pathways with the most annotated differential metabolites were: ko01100 (metabolic pathways, 16 metabolites), ko01110 (Biosynthesis of secondary metabolites, 15 metabolites), ko00564 (glycerophospholipid metabolism, nine metabolites), ko00590 (Arachidonic acid metabolism, four metabolites), and ko00591 (Linoleic acid metabolism, six metabolites), as shown in Table 3. Pathway enrichment analysis showed that five pathways were significantly enriched (*p* < 0.05), including glycerophospholipid metabolism (ko00564), Linoleic acid metabolism (ko00591), alpha-linolenic acid metabolism (ko00592), Arachidonic acid metabolism (ko00590), and Biosynthesis of secondary metabolites (ko01110) (Figure 11). Among these, glycerophospholipid metabolism had the highest enrichment degree (Rich Factor = 0.122), followed by Linoleic acid metabolism (Rich Factor = 0.194), as shown in Table 3.

**Figure 12:**
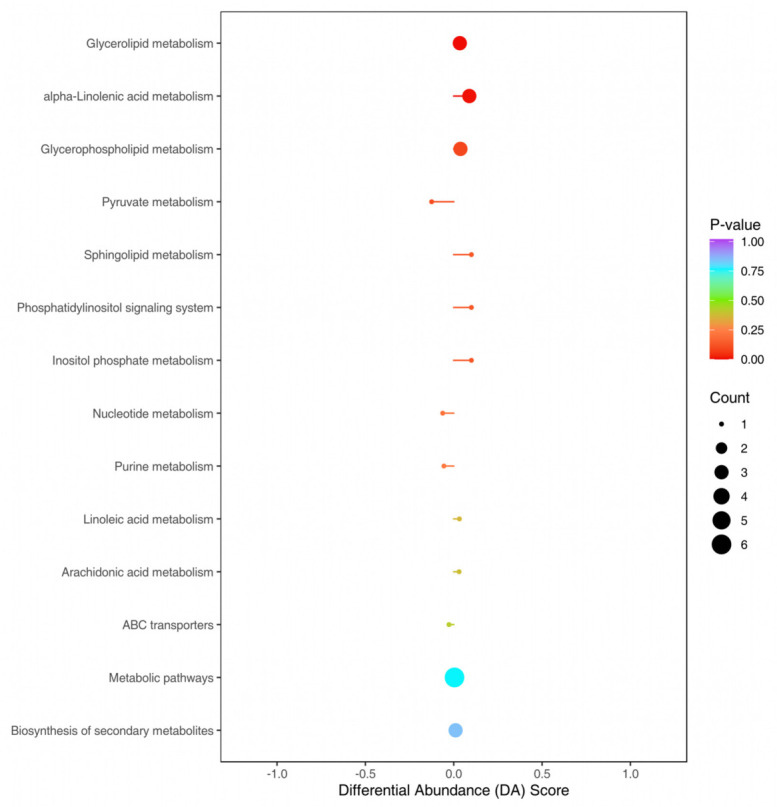
Pathway enrichment analysis based on differential abundance (DA) scores between MJH-EVs and MJY-EVs. The plot displays significantly enriched KEGG pathways. The x-axis represents the differential abundance (DA) score, reflecting the overall directional change in metabolites in each pathway between the two groups. The color of each pathway dot indicates the *p*-value (redder colors correspond to smaller *p*-values, indicating higher enrichment significance), and the size of the dot reflects the count of differential metabolites mapped to the pathway. The most significantly enriched pathways include glycerolipid metabolism, alpha-linolenic acid metabolism, and glycerophospholipid metabolism.

Differential abundance score (DA Score) analysis showed that three pathways had positive DA Scores, indicating overall upregulation, and two pathways had negative DA Scores, indicating overall downregulation (Figure 12). The pathway with the highest DA Score was Biosynthesis of secondary metabolites (DA Score = 0.047), followed by glycerophospholipid metabolism (DA Score = 0.027) and Linoleic acid metabolism (DA Score = 0.026), suggesting that these pathways are activated in flower-derived EVs. The pathways with negative DA Scores were Arachidonic acid metabolism (DA Score = −0.030) and alpha-linolenic acid metabolism (DA Score = −0.029), indicating that these pathways are more active in leaf-derived EVs.

#### 3.1.6. Analysis of Correlations and Functional Prediction of DMs

This network indicates the interconnectedness of tissue-specific DMs and highlights the central role of lipid metabolism in shaping the metabolic divergence between flower and leaf EVs (Figure 10, Figure 11 and Figure 12, Table 2, Supplementary Table S2). Correlation network analysis further revealed the interconnectedness of DMs (Figure 10, Figure 11 and Figure 12). Lipid-related classes (Fatty Acyls, glycerophospholipids) emerged as core nodes with high connectivity, supported by widespread positive correlations (pink edges) that may contribute to co-regulation within lipid metabolic networks. Class-specific clustering of metabolites also highlighted shared regulatory mechanisms across functionally related DMs, providing a system-level view of tissue-specific metabolic regulation.

Based on KEGG annotation and existing research reports, the functions of key differential metabolites were predicted as follows. Functional annotation of differential metabolites in MJH might participate in their involvement in multiple critical physiological processes of plants. Specifically, upregulated metabolites in MJH, including Cuminaldehyde (MEDP0603), have been reported to possess antioxidant activities, which may contribute to protecting flowers against oxidative stress during the reproductive stage. Additionally, 2-Hexenal (ME0112152) in MJH is implicated in plant stress response pathways, suggesting that flower-derived EVs might play a vital role in defending against both biotic and abiotic stresses (Figure 10, Figure 11 and Figure 12, Table 2, Supplementary Table S2). Furthermore, downregulated metabolites in MJH such as Palmitic amide (MEDP0512) and Valeric acid (ME0103073) may be associated with lipid synthesis and energy metabolism, processes that are more active in leaves to support photosynthetic efficiency. Notably, adenine (ME0105438) and arginine (ME0159989), which participate in purine metabolism and amino acid metabolism, respectively, may be involved in intercellular signal transduction across different plant tissues (Figure 10, Figure 11 and Figure 12, Table 2, Supplementary Table S2).

## 4. Discussion

Plant extracellular vesicles (EVs) are key regulators of intercellular communication, and their metabolic cargo closely reflects the physiological functions of source tissues [1,22]. As a medicinal and ornamental plant with significant economic and ecological value, Hibiscus syriacus accumulates abundant bioactive metabolites in flowers and leaves; however, the tissue-specific metabolic features of its EVs remain unreported. In this study, we applied LC-MS/MS-based untargeted metabolomics to systematically profile EV metabolomes from *H. syriacus* flowers and leaves, identifying distinct tissue-specific metabolic signatures that align with organ functional differentiation. These results fill the critical knowledge gap regarding tissue-specific EV metabolism in *H. syriacus* and provide novel insights into the biological functions and application potential of plant-derived EVs.

Transmission electron microscopy (TEM) and dynamic light scattering (DLS) confirmed the successful isolation of EVs from *H. syriacus* flowers and leaves, with spherical or cup-shaped morphologies and a diameter range of 50–220 nm (Figure 1A–D). These structural features are highly consistent with the canonical characteristics of plant EVs reported in previous studies: for instance, EVs isolated from ginger exhibit a diameter range of 40–200 nm with spherical morphologies [23], while grape-derived EVs have a size distribution of 60–230 nm [24], and broccoli EVs range from 55 to 210 nm [25]. The slight difference in mean hydrodynamic diameter between MJH-EVs (75.2 ± 8.5 nm) and MJY-EVs (90 ± 29.0 nm) is not only statistically significant (Figure 1) but may also likely reflect intrinsic differences in membrane composition or biogenesis mechanisms between leaf and flower tissues. Consistent with our observation, previous studies have demonstrated that plant EV size and composition vary across tissues, reflecting functional specialization and distinct physiological roles [1,3]. Notably, the D90:D10 ratios (2.16–2.2) and span values (0.92–0.95) of *H. syriacus* EVs indicated a moderately homogeneous vesicle population, which is comparable to EVs isolated from other plant species (tomato EVs with span values of 0.88–1.02 [3], and soybean EVs with D90:D10 ratios of 2.0–2.3 [26]). This consistency not only validates the reliability of our isolation protocol but also ensures that the subsequent metabolomic analysis is based on high-quality, representative EV samples, avoiding potential biases caused by heterogeneous vesicle populations. Furthermore, there is an absence of obvious impurities in TEM images (Figure 1A,C).

Global metabolomic profiling identified 3338 metabolites across EV samples, dominated by lipids and lipid-like molecules (29.43%), followed by organic acids (17.98%) and organoheterocyclic compounds (14.21%) (Figure 2 and Figure 3). This observation is consistent with previous findings that plant EVs are inherently enriched in lipids, which form the structural basis of their lipid bilayer and mediate critical biological processes such as cargo encapsulation, intercellular transport, and membrane fusion [27]. Importantly, the lipid content of *H. syriacus* EVs (29.43%) is slightly higher than that of ginger EVs (27.1%) and grape EVs (26.8%) [28], which may be related to the high lipid synthesis capacity of *H. syriacus* tissues—consistent with previous reports that *H. syriacus* leaves accumulate abundant membrane lipids to support efficient photosynthesis [11]. The predominance of organic acids and derivatives (17.98%) and organoheterocyclic compounds (14.21%) in *H. syriacus* EVs further reflects their unique metabolic diversity compared to EVs from other plant species. For example, EVs from Arabidopsis thaliana have a lower proportion of organic acids (12.3%) [29], while those from Medicago truncatula have a higher proportion of organoheterocyclic compounds (16.5%) but lower lipids (24.1%) [30]. This difference may be attributed to the specific physiological characteristics of *H. syriacus*: organic acids are key intermediates in the tricarboxylic acid cycle (TCA cycle), which provides energy for EV biogenesis and cargo transport, while organoheterocyclic compounds (flavonoids, alkaloids) are important secondary metabolites involved in plant defense and environmental adaptation [10].

Multivariate statistical analysis (PCA and OPLS-DA) revealed clear separation between MJY-EVs and MJH-EVs (Figure 4A,B), with the OPLS-DA model exhibiting high robustness (R^2^Y = 0.929, Q^2^ = 0.852) (Figure 5); a Q^2^ value greater than 0.8 indicates that the model has excellent predictive ability, which is higher than the Q^2^ values reported in similar studies (Q^2^ = 0.78 for Arabidopsis EVs, Q^2^ = 0.81 for grape EVs [8]). This distinct clustering is indicative of strong tissue-specific metabolic divergence, which tightly aligns with the functional differentiation of plant tissues: leaves serve as the primary site of photosynthesis and material synthesis, while flowers are specialized for reproduction and stress resistance [11]. Similar tissue-specific metabolic signatures have been reported in Arabidopsis thaliana and Medicago truncatula [30], but our study extends this observation to EVs from a non-model medicinal plant, highlighting that EV metabolic cargo is universally coupled to the physiological functions of their source tissues, regardless of plant species.

Metabolomic profiling of extracellular vesicles (EVs) from *H. syriacus* flower and leaf tissues revealed a functionally specialized cargo dominated by lipids and lipid-like molecules (29.43%), followed by organic acids and derivatives (17.98%), organoheterocyclic compounds (14.21%), and benzenoids (12.71%) as shown in Figure 6, which extends our understanding of plant EVs beyond model species, demonstrating that these vesicles act as selective carriers of structural, signaling, and defensive metabolites in ornamental plants. The predominance of bioactive lipids (oxylipins) supports a role in systemic stress signaling, while organic acids reflect energetic demands and redox homeostasis, and benzenoids/phenylpropanoids underscore contributions to UV protection, pathogen defense, and cell wall reinforcement [31,32]. A total of 39 differential metabolites (DMs) were identified between MJY-EVs and MJH-EVs (VIP > 1, *p* < 0.05), with 31 upregulated in MJH-EVs and eight upregulated in MJY-EVs (Figure 7, Supplementary Table S2). This 4:1 ratio of upregulated to downregulated metabolites in leaf-derived EVs suggests a marked bias toward metabolic activation associated with leaf-specific physiological processes.

The top DMs with the highest VIP values included propamocarb (VIP = 2.11), maclurin (VIP = 2.08), and pentadecanoic acid (VIP = 1.99) (Table 2), which are closely involved in lipid metabolism and secondary metabolism—processes critical for flower development and antioxidant defense. Notably, MJH-EVs were significantly enriched in lipids and lipid-like molecules, such as pentadecanoic acid (log_2_FC = 4.61, *p* < 0.001), 1-palmitoylglycerol 3-phosphate (log_2_FC = 3.26, *p* < 0.01), and lysophosphatidic acid (log_2_FC = 3.03, *p* < 0.01) (Supplementary Table S2). These metabolites are key intermediates in glycerophospholipid metabolism (ko00564), which was the most significantly enriched pathway (Rich Factor = 0.122, *p* < 0.001) (Table 3, Figure 11)—with a Rich Factor higher than that reported in ginger EVs (0.105) [1] and soybean EVs (0.111) [26]. Glycerophospholipids are major components of photosynthetic membranes (thylakoid membranes), accounting for approximately 60–70% of the total membrane lipids in plant leaves [17]. Their significant enrichment in leaf EVs is consistent with the role of leaves in photosynthesis and membrane biogenesis: pentadecanoic acid, a C15 odd-chain saturated fatty acid synthesized via α-oxidation, contributes to lipid pool maintenance and membrane stability, which are essential for sustaining photosynthetic activity under environmental fluctuations (drought, high temperature) [33]. This finding strongly supports our hypothesis that leaf-derived EVs prioritize lipid metabolism to support photosynthetic membrane function and leaf stress tolerance.

In contrast, MJY-EVs were enriched in metabolites involved in nucleotide metabolism and secondary biosynthesis, such as inosine (log_2_FC = −1.95, *p* < 0.01) and acetylenedicarboxylic acid (log_2_FC = −2.21, *p* < 0.01) (Figure 8). Inosine, a nucleoside derivative, participates in purine salvage pathways to meet the high energy demand of floral reproductive processes (pollen development, petal expansion) [34]—consistent with previous reports that *H. syriacus* flowers have high energy metabolism activity during blooming [16]. Acetylenedicarboxylic acid, a key intermediate in secondary metabolism, may regulate the synthesis of flower-specific secondary metabolites (anthocyanins, flavonoids) that are associated with flower pigmentation and defense against pathogens [10]. These observations align with previous reports that *H. syriacus* flowers are rich in bioactive secondary metabolites with medicinal properties, such as anthocyanins and organic acids [10,11], further confirming that flower-derived EVs act as carriers of these functional metabolites, potentially facilitating their transport within floral tissues or to other plant organs.

Heatmap and pathway analyses further confirmed that lipids are the primary drivers of EV metabolic divergence, with MJH-EVs clustering with lipid derivatives linked to stress signaling and MJY-EVs associating with purine and organic acid metabolism related to energy supply. For example, upregulated pentadecanoic acid and lysophosphatidic acid in floral EVs clustered with other lipid derivatives, reinforcing the hypothesis that floral EVs specialize in lipid-mediated stress signaling. In contrast, foliar EVs exhibited higher levels of purine metabolites (inosine) and organic acids, aligning with their role as source tissues supporting energetic metabolism. Notably, the clear separation of floral and foliar EV metabolic profiles in the heatmap provides direct evidence of tissue-specific EV cargo sorting, a phenomenon rarely documented in ornamental species [35]. KEGG pathway enrichment analysis (Figure 10) revealed that 60% of annotated metabolites mapped to metabolic pathways, with glycerolipid metabolism, glycerophospholipid metabolism, and alpha-linolenic acid metabolism each accounting for 30% of this category. This dominance of lipid metabolic pathways aligns with the structural definition of EVs as lipid-bilayer-enclosed vesicles, but further highlights a functional bias toward membrane homeostasis and lipid signaling. For example, glycerophospholipid metabolism generates lysophosphatidic acid (LPA)—a key lipid second messenger linked to plant stress responses and cell proliferation [36]—while alpha-linolenic acid metabolism produces jasmonic acid (JA) precursors, which mediate systemic defense signaling [37]. The enrichment of organismal system pathways (20%), including vitamin and fat digestion and absorption, also suggests EVs may facilitate nutrient transport between source (leaf) and sink (flower) tissues, supporting floral development and senescence resilience. Notably, polyphenols such as anthocyanins were not detected in *H. syriacus* EVs, likely due to their high polarity, instability during LC-MS/MS analysis, or selective exclusion during EV biogenesis. This observation aligns with the general preference of plant EVs for lipids and moderately polar metabolites, suggesting that EV cargo sorting is governed by membrane lipid composition and endosomal trafficking machineries—mechanisms that require further investigation.

KEGG pathway enrichment analysis further confirmed that lipid metabolism pathways (glycerophospholipid, linoleic acid, and α-linolenic acid metabolism) were the most significantly enriched (Figure 11), which is consistent with the predominance of lipid DMs. Previous studies have demonstrated that plant EVs mediate intercellular communication via lipid signaling [18], and our results extend this by showing that tissue-specific lipid metabolic networks may underpin distinct EV functions: leaf EVs facilitating membrane maintenance and stress response, and flower EVs supporting reproduction and secondary metabolism. Additionally, the enrichment of purine metabolism in leaf EVs (via adenine) provides energy substrates for leaf growth and photosynthesis, while the enrichment of secondary metabolite biosynthesis in flower EVs may enhance floral fitness and defense against pathogens [18]. Notably, the correlation analysis of DMs (Figure 12) revealed a strong positive correlation between pentadecanoic acid and 1-palmitoylglycerol 3-phosphate (r = 0.89, *p* < 0.001), indicating a coordinated regulation of glycerophospholipid metabolism in leaf EVs, which provides a system-level view of EV metabolic regulation.

In summary, this study systematically characterized the metabolomic profiles of *H. syriacus* flower and leaf EVs using LC-MS/MS-based untargeted metabolomics, combined with multivariate statistical analysis and pathway enrichment analysis, identifying distinct tissue-specific metabolic signatures. Notably, polyphenols, including anthocyanins, were not detected in EVs. This is likely due to their high polarity, instability under UPLC conditions, and low abundance in EV cargo, consistent with the preferential enrichment of lipids and moderately polar metabolites in plant EVs. Therefore, tissue-specific cargo sorting may be driven by membrane lipid composition and endosomal sorting complexes required for EV biogenesis, which warrants further investigation. These findings support applied uses, with flower EVs being suitable for cosmetics (antioxidant) and leaf EVs for agriculture (stress tolerance).

## 5. Conclusions

Collectively, these findings suggest that Hibiscus syriacus EVs exhibit tissue-specific metabolic specialization, with MJH-EVs prioritizing lipid metabolism for photosynthetic and stress-related functions and MJY-EVs focusing on secondary metabolite and nucleotide metabolism for reproduction and defense—providing a theoretical basis for targeted applications of plant-derived EVs in cosmetics, agriculture, and natural product development.

## Figures and Tables

**Table 1:** Internal standard information and its stability in QC samples.

Index	Q1 Molecular Weight (Da)	RT (min)	CV	Name	CAS. No	Purity	Company
MWS201357-IS-P	403.3601	4.69	0.0239	CAR(16:0)-d3	202480-73-7	>98%	Sigma (St. Louis, MO, USA)
MWS3085-IS-P	281.0048	3.78	0.0339	D-Luciferin	2591-17-5	>98%	Rhawn (Louisville, KY, USA)
MWS20572-IS-P	198.0977	2.83	0.0465	caffeine 13C3	58-08-2	>98%	Chembind LLC (Atlanta, GA, USA)
MWS04187-IS-P	210.1282	2.21	0.0498	L-Tryptophan-d5	62595-11-3	>98%	isoreag (Shanghai, China)

**Table 2:** Top 10 differential metabolites with the highest VIP values.

Index	Compounds	VIP	*p*-Value	Log2FC (MJH/MJY)	Type
ME0169706	Propamocarb	2.11	0.00	0.64	up
ME0007535	Maclurin	2.08	0.01	2.17	up
ME0108343	Monoethyl carbonate	1.99	0.03	1.81	up
ME0061027	Pentadecanoic acid	1.99	0.04	4.61	up
ME0007146	Fesoterodine	1.98	0.00	−2.16	down
ME0110395	2-Pentanamidoacetic acid	1.97	0.00	1.61	up
ME0141551	17betHydroxy-2alph(methoxymethyl)- 17-methyl-5alphandrostan-3-one	1.96	0.00	2.19	up
ME0009496	Sodium polystyrene sulfonate	1.96	0.03	2.83	up
ME0012348	(9Z,11E,13S,15Z)-13-Hydroxyoctadec- 9,11,15-trienoic acid	1.95	0.02	2.65	up
ME0139112	Narirutin	1.94	0.01	0.64	up

**Table 3:** Statistics of differential metabolites annotated in KEGG pathways.

ko_ID	Pathway Name	Number of Annotated Differential Metabolites	Total Number of Annotated Metabolites in Pathway
ko01100	Metabolic pathways	16	440
ko01110	Biosynthesis of secondary metabolites	15	276
ko00564	Glycerophospholipid metabolism	9	74
ko00590	Arachidonic acid metabolism	4	33
ko00591	Linoleic acid metabolism	6	31
ko00592	Alpha-linolenic acid metabolism	6	34
ko00440	Phosphonate and phosphinate metabolism	1	8
ko00999	Biosynthesis of various plant secondary metabolites	1	24
ko00960	Tropane, piperidine and pyridine alkaloid biosynthesis	2	15
ko00561	Glycerolipid metabolism	3	26

## Data Availability

The raw metabolomic data generated by LC-MS/MS in this study, including peak tables and metabolite annotation files, are available from the corresponding authors (Junhe Hu andYong Chen) upon reasonable request. All analytical software and databases used in the study are publicly available, as indicated in Section 2.

## Supplementary Materials

The following supporting information can be downloaded at: https://www.mdpi.com/article/10.3390/metabo16060386/s1, Table S1: Statistics of differential metabolites between MJH and MJY group, Table S2: the confident annotation of 237 unique metabolites between MJH and MJY group).

## References

[1] Ambrosone A., Barbulova A., Cappetta E., Cillo F., De Palma M., Ruocco M., Pocsfalvi G. (2023). Plant Extracellular Vesicles: Current Landscape and Future Directions. Plants.

[2] Cai Q., Halilovic L., Shi T., Chen A., He B., Wu H., Jin H. (2023). Extracellular Vesicles: Cross-Organismal RNA Trafficking in Plants, Microbes, and Mammalian Cells. Extracell. Vesicles Circ. Nucl. Acids.

[3] Bai C., Liu J., Zhang X., Li Y., Qin Q., Song H., Yuan C., Huang Z. (2024). Research Status and Challenges of Plant-Derived Exosome-like Nanoparticles. Biomed. Pharmacother..

[4] Wu R., Chen B., Jia J., Liu J. (2024). Relationship between Protein, MicroRNA Expression in Extracellular Vesicles and Rice Seed Vigor. Int. J. Mol. Sci..

[5] Mu N., Li J., Zeng L., You J., Li R., Qin A., Liu X., Yan F., Zhou Z. (2023). Plant-Derived Exosome-Like Nanovesicles: Current Progress and Prospects. Int. J. Nanomed..

[6] Orefice N.S., Di Raimo R., Mizzoni D., Logozzi M., Fais S. (2023). Purposing plant-derived exosomes-like nanovesicles for drug delivery: Patents and literature review. Expert Opin. Ther. Pat..

[7] Hong R., Luo L., Wang L., Hu Z.-L., Yin Q.-R., Li M., Gu B., Wang B., Zhuang T., Zhang X.-Y. (2023). Lepidium meyenii Walp (Maca)-derived extracellular vesicles ameliorate depression by promoting 5-HT synthesis via the modulation of gut-brain axis. Imeta.

[8] Perut F., Roncuzzi L., Avnet S., Massa A., Zini N., Sabbadini S., Giampieri F., Mezzetti B., Baldini N. (2021). Strawberry-Derived Exosome-Like Nanoparticles Prevent Oxidative Stress in Human Mesenchymal Stromal Cells. Biomolecules.

[9] Chew L.Y., Teng S.K., Neo Y.P., Sim Y.Y., Chew S.C. (2024). The Potential of Roselle (*Hibiscus sabdariffa*) Plant in Industrial Applications: A Promising Source of Functional Compounds. J. Oleo Sci..

[10] Liu H., Wang X., Li A., Zhang L., Mo Q., Zhang S. (2025). Organic Acids Copigmentation With *Hibiscus Syriacus* Flower (*Hibiscus syriacus* L.) Anthocyanins: Thermal Stability, Characterization, and Copigmentation Mechanisms. J. Food Sci..

[11] Xu X., Du K., Zhang C., Zheng C., Wang L., Wang Y., Meng D. (2026). *Hibiscus syriacus* L. Flower Attenuates Ethanol-Induced Gastric Ulcer via Modulation of the Nrf2/HO-1, AKT, and JNK Pathways. J. Ethnopharmacol..

[12] Chiu C.-T., Chen J.-H., Chou F.-P., Lin H.-H. (2015). Hibiscus sabdariffa Leaf Extract Inhibits Human Prostate Cancer Cell Invasion via Down-Regulation of Akt/NF-kB/MMP-9 Pathway. Nutrients.

[13] Tahmasebi A., Ebrahimie E., Pakniyat H., Ebrahimi M., Mohammadi-Dehcheshmeh M. (2019). Tissue-Specific Transcriptional Biomarkers in Medicinal Plants: Application of Large-Scale Meta-Analysis and Computational Systems Biology. Gene.

[14] Manickam S., Rajagopalan V.R., Kambale R., Rajasekaran R., Kanagarajan S., Muthurajan R. (2023). Plant Metabolomics: Current Initiatives and Future Prospects. Curr. Issues Mol. Biol..

[15] Yu X., Sun H., Gao X., Zhang C., Sun Y., Wang H., Zhang H., Shi Y., He X. (2022). A Comprehensive Analysis of Age-Related Metabolomics and Transcriptomics Reveals Metabolic Alterations in Rat Bone Marrow Mesenchymal Stem Cells. Aging.

[16] Wang X., Li L., Liu C., Zhang M., Wen Y. (2022). An Integrated Metabolome and Transcriptome Analysis of the *Hibiscus syriacus* L. Petals Reveal the Molecular Mechanisms of Anthocyanin Accumulation. Front. Genet..

[17] Ou Y., Teng Z., Shu Y., Wang Y., Wang D., Sun C., Lin X. (2025). Linoleic Acid Alleviates Aluminum Toxicity by Modulating Fatty Acid Composition and Redox Homeostasis in Wheat (*Triticum aestivum*) Seedlings. J. Hazard. Mater..

[18] Rutter B.D., Innes R.W. (2018). Extracellular Vesicles as Key Mediators of Plant–Microbe Interactions. Curr. Opin. Plant Biol..

[19] Kankaanpää S., Väisänen E., Goeminne G., Soliymani R., Desmet S., Samoylenko A., Vainio S., Wingsle G., Boerjan W., Vanholme R. (2024). Extracellular Vesicles of Norway Spruce Contain Precursors and Enzymes for Lignin Formation and Salicylic Acid. Plant Physiol..

[20] Lee B.-H., Wu S.-C., Chien H.-Y., Shen T.-L., Hsu W.-H. (2023). Tomato-Fruit-Derived Extracellular Vesicles Inhibit *Fusobacterium Nucleatum via* Lipid-Mediated Mechanism. Food Funct..

[21] Lo K.-J., Wang M.-H., Ho C.-T., Pan M.-H. (2024). Plant-Derived Extracellular Vesicles: A New Revolutionization of Modern Healthy Diets and Biomedical Applications. J. Agric. Food Chem..

[22] Wang R., Zhang Y., Guo Y., Zeng W., Li J., Wu J., Li N., Zhu A., Li J., Di L. (2025). Plant-Derived Nanovesicles: Promising Therapeutics and Drug Delivery Nanoplatforms for Brain Disorders. Fundam. Res..

[23] Zhu H., He W. (2023). Ginger: A Representative Material of Herb-Derived Exosome-like Nanoparticles. Front. Nutr..

[24] Castelli G., Logozzi M., Mizzoni D., Di Raimo R., Cerio A., Dolo V., Pasquini L., Screnci M., Ottone T., Testa U. (2023). Ex Vivo Anti-Leukemic Effect of Exosome-like Grapefruit-Derived Nanovesicles from Organic Farming-The Potential Role of Ascorbic Acid. Int. J. Mol. Sci..

[25] Del Pozo-Acebo L., López De Las Hazas M.-C., Tomé-Carneiro J., Del Saz-Lara A., Gil-Zamorano J., Balaguer L., Chapado L.A., Busto R., Visioli F., Dávalos A. (2022). Therapeutic Potential of Broccoli-Derived Extracellular Vesicles as Nanocarriers of Exogenous miRNAs. Pharmacol. Res..

[26] Dad H.A., Gu T.-W., Zhu A.-Q., Huang L.-Q., Peng L.-H. (2021). Plant Exosome-like Nanovesicles: Emerging Therapeutics and Drug Delivery Nanoplatforms. Mol. Ther..

[27] Savcı Y., Kırbaş O.K., Bozkurt B.T., Abdik E.A., Taşlı P.N., Şahin F., Abdik H. (2021). Grapefruit-Derived Extracellular Vesicles as a Promising Cell-Free Therapeutic Tool for Wound Healing. Food Funct..

[28] Karamanidou T., Tsouknidas A. (2021). Plant-Derived Extracellular Vesicles as Therapeutic Nanocarriers. Int. J. Mol. Sci..

[29] Jokhio S., Peng I., Peng C.-A. (2024). Extracellular Vesicles Isolated from Arabidopsis Thaliana Leaves Reveal Characteristics of Mammalian Exosomes. Protoplasma.

[30] Kranawetter C., Zeng S., Joshi T., Sumner L.W. (2021). A Medicago Truncatula Metabolite Atlas Enables the Visualization of Differential Accumulation of Metabolites in Root Tissues. Metabolites.

[31] Li Z., Liu D., Wang D., Sun M., Zhang G., Wu Y., Zhang Y., Cheng B. (2024). Study on the Causes of Changes in Colour during *Hibiscus syriacus* Flowering Based on Transcriptome and Metabolome Analyses. BMC Plant Biol..

[32] Choi Y., Park Y.H., Yang C., Kim D.H., Lee K.W., Lee M.Y. (2023). Protocol for a Randomized Controlled Trial Evaluating the Effect of *Hibiscus syriacus* L. Flower Extract on Sleep Quality. Front. Nutr..

[33] Venn-Watson S., Lumpkin R., Dennis E.A. (2020). Efficacy of Dietary Odd-Chain Saturated Fatty Acid Pentadecanoic Acid Parallels Broad Associated Health Benefits in Humans: Could It Be Essential?. Sci. Rep..

[34] Kim I.S., Jo E.-K. (2022). Inosine: A Bioactive Metabolite with Multimodal Actions in Human Diseases. Front. Pharmacol..

[35] Liu G., Kang G., Wang S., Huang Y., Cai Q. (2021). Extracellular Vesicles: Emerging Players in Plant Defense Against Pathogens. Front. Plant Sci..

[36] Zhang W.J., Zhou Y., Zhang Y., Su Y.H., Xu T. (2023). Protein Phosphorylation: A Molecular Switch in Plant Signaling. Cell Rep..

[37] Wasternack C., Hause B. (2013). Jasmonates: Biosynthesis, Perception, Signal Transduction and Action in Plant Stress Response, Growth and Development. An Update to the 2007 Review in Annals of Botany. Ann. Bot..

