# Supplementary material for: Metabolomic Profiling of Extracellular Vesicles from Flower and Leaf Tissues of Hibiscus syriacus
Source: Metabolites. 2026 Jun 2;16(6):386. doi: 10.3390/metabo16060386 (PMC13304267; doi:10.3390/metabo16060386)
Supplement: Supplementary file 1 [file metabolites-16-00386-s001.zip › Table S1.pdf]

Table S1 Statistics of differential metabolites between MJH and MJY group

Index	Compounds	Super.Class	Formula	MJH1	MJH2	MJH3	MJY1	MJY2	MJY3	Log2FC	Type
ME0061027	Pentadecanoic acid	Lipids and lipid-like molecules	C15H30O2	46695988	24312448	27631978	2841231	331012	862498	4.61	up
ME0107524	Imazamox	Organic acids and derivatives	C15H19N3O4	42381548	41557613	65795786	73221	5077115	6301324	3.71	up
ME0163991	1-Palmitoylglycerol 3-phosphate	Lipids and lipid-like molecules	C19H37O7P-2	130301089	132786769	107248404	1651264	23676396	13242291	3.26	up
ME0054488	Lysophosphatidic acid	Lipids and lipid-like molecules	C21H41O7P	319564570	470685116	418524187	5955784	60833365	80741881	3.03	up
ME0009496	Sodium polystyrene sulfonate	Benzenoids	C8H8O3S	35171866	19199537	30105317	2428105	7251722	2181019	2.83	up
ME0103160	x7-Hydroxyhexadecanedioic acid	Lipids and lipid-like molecules	C16H30O5	7975959	17628469	17486117	535174	315574	5645450	2.73	up
ME0012348	(9Z,11E,13S,15Z)-13-Hydroxyoctadec9,11,15-trienoic acid	Lipids and lipid-like molecules	C18H30O3	343391705	221472401	212223135	19969389	72745150	30737960	2.65	up
ME0055527	[(2R)-2-octanoyloxy-3-phosphonooxypropyl] decanoate	Lipids and lipid-like molecules	C21H41O8P	377255957	267113037	274383388	118754471	4001094	29620335	2.59	up
ME0125997	Oxazepam	Organoheterocyclic compounds	C15H11ClN2O2	67692939	42946588	38003075	17977647	4293428	9777084	2.21	up
ME0141551	17betHydroxy-2alph(methoxymethyl)-17 -methyl-5alphandrostan-3-one	Lipids and lipid-like molecules	C22H36O3	19533604	18826194	16626750	2149450	3302274	6573171	2.19	up
ME0015657	all-trans-Pentaprenyl diphosphate	Lipids and lipid-like molecules	C25H44O7P2	34190611	38275442	37743423	417083	8417046	15486339	2.18	up
ME0007535	Maclurin	Benzenoids	C13H10O6	69254351	64209567	52706344	10598954	15140131	15597396	2.17	up
ME0125244	Mycophenolate mofetil	Organoheterocyclic compounds	C23H31NO7	3817214	5041810	5613488	58753	3193969	419424	1.98	up
ME0015395	9-Hydroxy-10,12,15-octadecatrienoic acid	Lipids and lipid-like molecules	C18H30O3	113207589	172038384	199321494	19956067	73931632	31876598	1.95	up
ME0154660	Nupharamine	Alkaloids and derivatives	C15H25NO2	5942629	8383984	11810770	4691660	1333324	1175791	1.86	up
ME0108343	Monoethyl carbonate	Organic acids and derivatives	C3H6O3	135865062	209919999	227290893	69062534	53299052	40562621	1.81	up
ME0057831	PC(O-16:0_22:6)	Lipids and lipid-like molecules	C46H82NO7P	7835652	13922100	14066309	2609736	5318911	2270226	1.81	up
ME0055369	Norethindrone acetate	Lipids and lipid-like molecules	C22H28O3	5298930909	6366703685	9823741844	317703336	3754642959	2146815473	1.79	up
ME0055241	N-acetylsphingosine	Lipids and lipid-like molecules	C20H39NO3	125858422	113062959	119411612	979701	41476119	63457436	1.76	up
ME0110395	2-Pentanamidoacetic acid	Organic acids and derivatives	C7H13NO3	29105524	33071900	33858816	6690820	14600748	10093554	1.61	up
ME0112719	Gadusol	Organic oxygen compounds	C8H12O6	867616	1383357	1413740	584507	392861	263011	1.56	up
ME0105268	2-n-Propyl-4-oxopentanoic acid	Organic acids and derivatives	C8H14O3	51009622	66532174	62668177	12526318	12549528	37688233	1.52	up
ME0108433	N-(3-Oxohexanoyl) homoserine lactone	Organic acids and derivatives	C10H15NO4	19622788	29551593	30379629	2655190	17244012	8271045	1.50	up
ME0146828	Bornyl acetate	Lipids and lipid-like molecules	C12H20O2	15950792	21022491	22645955	1219609	9673718	11053493	1.44	up
ME0010883	(1R,3R,4R,5S,6S,8x)-1-Acetoxy-8-angeloyloxy-3,4-epoxy-5-hydroxy-7(14),10-bisaboladien-2-one	Lipids and lipid-like molecules	C22H30O7	26969458	18286089	24982143	15812848	5423922	5637508	1.39	up
ME0007768	Methyl benzoate	Benzenoids	C8H8O2	15076460	10691330	12742956	1984502	7176778	6767537	1.27	up
ME0106224	Cyclohexyl formate	Organic acids and derivatives	C7H12O2	64965123	44313812	55505997	21831277	13144053	34255313	1.25	up
MEDL01767	Damascenone	Organic oxygen compounds	C13H18O	9003961	10710715	9221272	4760200	6238392	2734364	1.08	up
ME0012382	Isopalmitic acid	Lipids and lipid-like molecules	C16H32O2	67892689	53252601	61511764	46451810	25581375	25105739	0.91	up
ME0139112	Narirutin	Phenylpropanoids and polyketides	C27H32O14	15101195	20043646	17139932	7733153	11200355	9185414	0.89	up
ME0169706	Propamocarb	Organic acids and derivatives	C9H20N2O2	997703	1028688	1049871	668202	654889	654870	0.64	up
ME0160561	Levulinic acid	Organic acids and derivatives	C5H8O3	21063956	15330138	20966067	28593132	30066584	23163318	-0.51	down
ME0054335	Lewis A trisaccharide	Organic oxygen compounds	C20H35NO15	5421046	2442415	2044829	7960767	12411841	11945679	-1.71	down
ME0123152	Brassilexin	Organoheterocyclic compounds	C9H6N2S	2553976	7182099	7750600	25453459	13308978	24115508	-1.85	down
ME0159913	Inosine	Nucleosides, nucleotides, and analogues	C10H12N4O5	22377041	11199855	10922913	68034447	36223679	67838729	-1.95	down
ME0013400	2-Arachidonyl Glycerol ether	Lipids and lipid-like molecules	C23H40O3	475396	375663	124389	1890725	1429679	1015890	-2.15	down
ME0007146	Fesoterodine	Benzenoids	C26H37NO3	1626151	600837	870352	4719215	4732605	4378764	-2.16	down
ME0148463	Fosfestrol	Phenylpropanoids and polyketides	C18H22O8P2	265990	10167259	7147737	25106669	36469138	19019656	-2.20	down
ME0105420	Acetylenedicarboxylic acid	Organic acids and derivatives	C4H2O4	47577516	12340522	12392616	83387412	147646121	102689092	-2.21	down
